# A novel animal model for skin flap prelamination with biomaterials

**DOI:** 10.1038/srep34144

**Published:** 2016-09-23

**Authors:** Xianyu Zhou, Xusong Luo, Fei Liu, Chuan Gu, Xi Wang, Qun Yang, Yunliang Qian, Jun Yang

**Affiliations:** 1 Department of Plastic and Reconstructive Surgery, Shanghai 9th People’s Hospital, Shanghai Jiaotong University School of Medicine, Shanghai, 200011, China

## Abstract

Several animal models of skin flap construction were reported using biomaterials in a way similar to prefabrication. However, there are few animal model using biomaterials similar to prelamination, another main way of clinical skin flap construction that has been proved to be reliable. Can biomaterials be added in skin flap prelamination to reduce the use of autogenous tissues? Beside individual clinical attempts, animal model is needed for randomized controlled trial to objectively evaluate the feasibility and further investigation. Combining human Acellular Dermal Matrix (hADM) and autologous skin graft, we prelaminated flaps based on inguinal fascia. One, two, three and four weeks later, hADM exhibited a sound revascularization and host cell infiltration. Prelaminated skin flaps were then raised and microsurgically transplanted back to groin region. Except for flaps after one week of prelamination, flaps from other subgroups successfully reconstructed defects. After six to sixteen weeks of transplantation, hADM was proved to being able to maintain its original structure, having a wealth of host tissue cells and achieving full revascularization.To our knowledge, this is the first animal model of prelaminating skin flap with biomaterials. Success of this animal model indicates that novel flap prelamination with biomaterials is feasible.

For extensive composite defects or deformities, skin flap is often preferable choice for durable coverage or organ reconstruction^1^,^2^. However, the adequate source for skin flap harvesting is not necessarily available in patients with extensive trauma. Moreover, skin flap harvesting is associated with donor site morbidity, which may be even less tolerable for the patients. For these cases the treatment remains extremely challenging. There is not yet a satisfactory technology to tackle this problem.

Inspired by regenerative medicine^3^,^4^, one promising approach to address this challenge is the introduction of biomaterials to autogenic tissue for the generation of a novel skin flap. Such “on demand” construction would greatly reduce the need for autologous tissue and limit donor site morbidity.

Clinically, the two main ways of constructing flaps are prefabrication and prelamination^5^,^6^. Similarly, the addition of biomaterials can also be categorized as either prefabrication (relocate blood vessels to link biomaterials) or prelamination (adding biomaterials to autologous tissue based on existing blood vessels). Several skin flap prefabrication experiments using biomaterials in animals or human have been reported^7^,^8^,^9^; however, there are limited publications about skin flap prelamination with biomaterials. This is an area which warrants further investigation, as prelamination has been shown to be rapid and reliable technique associated with neovascularization.

Attempting to bridge this gap, we have developed a new animal model of skin flap prelamination using human Acellular Dermal Matrix (hADM) (Figs 1 and 2), a common biomaterial that has previously been used in other prefabrication experiments^10^. Here, we investigate the feasibility and reliability of this prelaminated skin flap.

## Results

### Skin graft take and hADM histopathological assessment throughout skin flap prelamination

On gross examination, slight ulceration was observed at the skin graft margin of the prelamination group at the first two postoperative weeks; however, these healed spontaneously, something which was not apparent in the control group. At postoperative week four, skin graft size was 7.7 ± 1.25 cm^2^ in the prelamination group and 7.4 ± 1.01 cm^2^ in the control group. There was no significant difference (p > 0.05). Skin grafts in both groups appeared normal in color, with soft texture and good elasticity (Fig. 3).

Histopathological analysis of hADM revealed full-thickness infiltration by cells, the majority of which were initially inflammatory cells and gradually replaced by fibroblasts. Luminal spaces lined by endothelial cells signified the formation of blood vessels, yet this process of neovascularization was slower to occur in the prelamination group. The average density of newly formed vessels in the prelamination group was 3.08 ± 1.29, 5.50 ± 1.50, 11.45 ± 4.24, 16.20 ± 4.68 at the prelamination times of one, two, three, four weeks respectively; this was compared to 5.63 ± 1.98, 7.68 ± 2.40, 14.25 ± 4.32, 16.03 ± 6.05 in the control group. There was significant difference (p ≤ 0.05) in blood vessel formation between the two groups before week three, but the prelamination group achieved almost same level of neovascularization as the control group at week four (p > 0.05). Fibroblastic proliferation and neovascularization were found throughout the full thickness of hADM at week two after implantation, which become quite apparent from week three. H&E and Masson’s trichrome staining showed that hADM maintained its original structure at four weeks following implantation.

### Survival of free prelaminated flap and histopathology evaluation of hADM long-term outcome

Only one week of prelamination failed to form sufficient vascular networks in the new skin flaps, which subsequently failed after microsurgical transplantation. Other prelamination subgroups (prelamination times of 2, 3, and 4 weeks) and all control subgroups survived and successfully reconstructed the original tissue deficit. Skin grafts which were prelaminated for 2 weeks prior to transplantation showed spot necrosis of skin at the flap margin, but healed spontaneously (Figs 4 and 5).

Biopsies were harvested from various subgroups, including 2 weeks of prelamination plus 6 weeks post-transplantation, 3 weeks of prelamination plus 12 weeks post-transplantation, and 4 weeks of prelamination plus 16 weeks post-transplantation. Histopathological evaluation demonstrates that hADM achieves sufficient cell infiltration and blood vessel formation, and maintains its original structure without obvious degradation. Long term results suggest that hADM is ideal for skin flap prelamination because it promotes superior neovascularization, maintains its original architecture, and incorporates into host tissue well to reach satisfactory reconstruction.

## Discussion

The emerging field of regenerative medicine has rendered possible the bioengineering of a skin flap, one type of engineered vascularized composite tissue (EVCT). The introduction of biomaterials into autologous tissues is a novel and effective modality for maximizing the efficiency of a patient’s own donor tissue and lowering the need for autografts. The engineered skin flap should recapitulate the physiologic and mechanical properties of a traditional skin flap, reduce donor site morbidity, and avoid the immunological hurdle of rejection faced by vascularized composite allografts^11^. The field of EVCT research is at the forefront of tissue engineering and reconstruction; lessons learned from enhancing native tissues may soon be applied to growing them de novo.

As shown in Table 1, research on the bioengineering of skin flaps to date mainly involves two aspects: the types of biomaterials used, and strategies for promoting neovascularization.

### Types of biomaterials

In order to form the main body of the skin flap, there are two kinds of biological materials: skin substitutes and bioprosthetic mesh^12^.

The main component of skin substitutes is animal-derived collagen which acts as a dermal analogue, such as porcine type I collagen and bovine collagen in Integra. Some skin substitutes are supplemented with live allogeneic cells (keratinocytes and/or fibroblasts) during manufacturing or autogeneic cells at the time of application. The strength of these substitutes is relatively weak and they are easy to contract. Currently, bioengineered skin substitutes are used most extensively in wound treatment, including for chronic venous and diabetic ulcers^13^.

Similar to skin substitutes, bioprosthetic meshes are derived from mammalian tissues. They are most commonly xenogeneic (such as Permacol, the porcine ADM) or can be allogeneic (such as AlloDerm). To manufacture these products, tissues are processed to remove cells and other potentially immunogenic components while preserving extracellular architecture into which host cells can grow and populate. Bioprosthetic mesh has a softer texture and greater mechanical strength than skin substitute. It is able to withstand larger physiological stresses, maintain its original size for longer periods of time, and is being increasingly used in the clinic to promote tissue regeneration in many reconstructive surgical procedures^14^,^15^.

### Strategies for promoting neovascularization

Transferred tissue cannot survive or regenerate without a functioning vasculature, and in clinical practice, there are two main ways of forming new blood vessels. The first is to isolate and place vessels on the biomaterials, or to form a bundle (arteriovenous unit) attached to biomaterials to form a viable axial flap. This is similar to flap prefabricaction. Another method is adding biomaterials to autologous tissue that already has rich blood supply; this is more consistent with flap prelamination, and refers to a two-stage process whereby biomaterials are engrafted into a reliable vascular bed to create a multilayered composite flap. This composite flap can be subsequently transferred en bloc on its original vascular supply for reconstruction.

Most previous studies (Table 1) chose prefabrication to achieve neovascularization. This method may be preferred by some over prelamination because the formation of new blood vessels from pedicle to biomaterial may be easily and directly visualized.

In the prelamination method of neovascularization, host tissue with a rich blood supply can be transferred following biomaterial incubation. This method ensures adequate vascular supply to the flap and leads to more consistent outcomes after reconstruction. Another advantage of the prelamination method of neovascularization is that this reliable blood supply and extensive contact area between biomaterial and host tissue may lead to shorter incubation time for biomaterials, which is especially important for bioengineering larger flaps.

Reconstructive surgeons frequently use arteries, such as the superficial temporal and cervical arteries, to provide vascular supply to large skin flaps. Instead of taking the artery alone, however, surgeons use the artery-containing fascia; this is very similar to the bioengineering concept of prelamination^16^,^17^.

The study at hand demonstrates that the inguinal fascia can provide sufficient blood supply to hADM and the skin flap. The rich blood supply of fascia makes it an excellent site for biomaterial implantation and tissue construction^18^. In addition, fascia has many advantages. Due to its deep anatomical location, fascia is often intact following trauma. For example, in severely burned patients with ear and periauricular skin loss, ear reconstruction will mainly depend on temporoparietal fascia^19^. Another advantage of fascia is that a wide range of axial fascia is available throughout the body, and many axial skin flaps can be reliably and flexibly raised as axial fascia-only flaps^20^,^21^. Furthermore, recent work shows that fascia itself is rich in stem cells (fascia derived stem cells, FDSCs), which possess multi-lineage differentiation potential and are responsive to the induction signals for collagen rich fascial structure regeneration^22^.

## Conclusion

There have been several clinical case reports which used biomaterial-involved prelaminated skin flaps for tissue reconstruction. Due to the unique, individualized nature of these injuries and reconstructions, and the difficulty in specimen harvests, however, it is difficult to design a clinical randomized control trial to objectively assess outcomes. Therefore, the establishment of a suitable animal model for assessing the prelamination of skin flaps with biomaterials prior to reconstruction is necessary.

To our knowledge, this is the first animal model using a skin flap prelaminated with biomaterials to reconstruct a large tissue defect. Our data suggests that hADM is an ideal biomaterial for skin flap prelamination: here we show that hADM achieves sufficient cell infiltration, neovascularization, and maintains its original structure without obvious degradation. We demonstrate that axial fascia can provide rich blood supply for biomaterial integration, and can form an axial skin flap which can be microsurgically transplanted for tissue reconstruction. The success of this animal model provides proof of principle that novel flap prelamination with biomaterials is feasible, and suggests it as a promising approach to reduce donor site morbidity and improve reconstructive outcomes. Future work on prelaminated skin flaps should investigate alternative types of biomaterials, seeding flaps with stem cells, and different flap construction techniques.

## Methods

### Animals

Adult male inbred Fisher 344 rats weighting 250 to 300 g were obtained from the Sino-British SIPPR/BK Lab Animal Ltd, Shanghai, China. Animals were maintained under standard conditions with food and water *ad libitum*.

### hADM

Commercialized human acellular dermal matrix (hADM) was purchased from Jie-Ya Life Biological Technology Co., Ltd (Beijing, China), and was approved by the China Food and Drug Administration for human use. A 2 cm × 3 cm plate of hADM with a median thickness of 1 mm was used as a xenogenic graft biomaterial (Fig. 2A,B).

### Prelamination surgery

**Figure 1:**
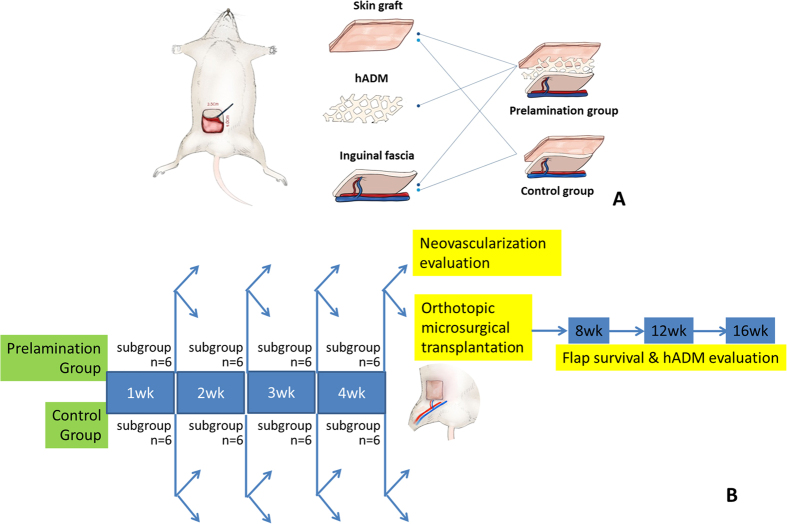
Flow chart of the experiment. (**A**) Using a rat model, control groups constructed a flap consisting of vascularized inguinal fascia and an overlying skin graft. Animals in the prelamination group constructed a similar flap with hADM between the two other layers. (**B**) Experimental protocol.

**Figure 2:**
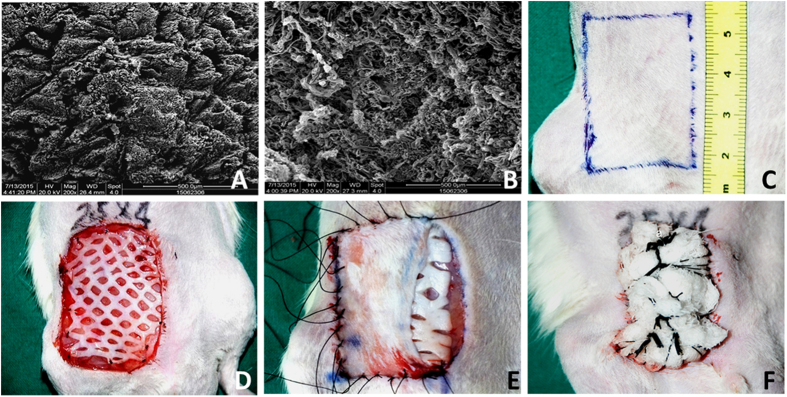
hADM and prelamination surgery. (**A**) Examination of hADM by scanning electron microscopy (SEM; QUANTA 200, FEI, Holland) showed a relatively looser meshwork of collagen fibers in the papillary layer, which appear to have niches for appendages. (**B**) SEM micrograph of the reticular layer of hADM exhibits a compact structure of collagen fibers whose architecture is continuous and relatively dense. (**C**) A 2.5 cm × 4.0 cm section was marked in the right inguinal region and resected to create a full thickness defect. (**D**) In experimental groups, meshed hADM was fixed on the inguinal fascia. (**E**) Split thickness skin graft harvested from previously resected skin was replanted to cover the hADM or inguinal fascia. (**F**) Transplanted hADM and skin graft were fixed with a bolster dressing.

**Figure 3:**
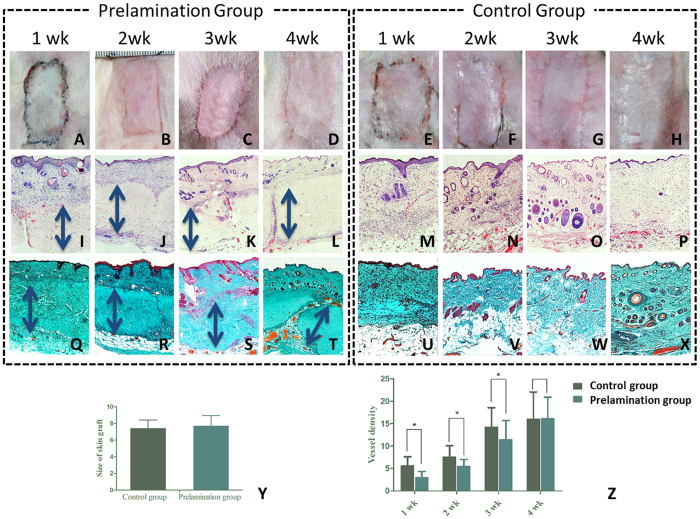
Skin graft take and hADM histopathological assessment throughout prelamination. (**A–D**) Gross images of skin grafts prelaminated with hADM at post-transplant weeks 1–4. (**E–H)** Gross images of control skin grafts at post-transplant weeks 1–4. (**I–L**) H&E staining of transplanted skin grafts in the prelamination group. hADM (double headed arrow) demonstrated increasing blood vessel formation and fibroblast infiltration. (**M–P**) H&E staining of transplanted skin graft controls. (**Q–T**) Masson’s trichrome of transplanted, prelamination skin grafts group. hADM (double head arrow) maintains its original structure *in vivo* at four weeks post-transplantation. (**U–X**) Masson’s trichrome of transplanted skin graft controls. (**Y**) comparison of prelaminated and control skin flap sizes at post-operative week four. (**Z**) comparison of blood vessel density between the hADM-skin graft interface in the prelamination group and fascia-skin graft interface in the control group.

**Figure 4:**
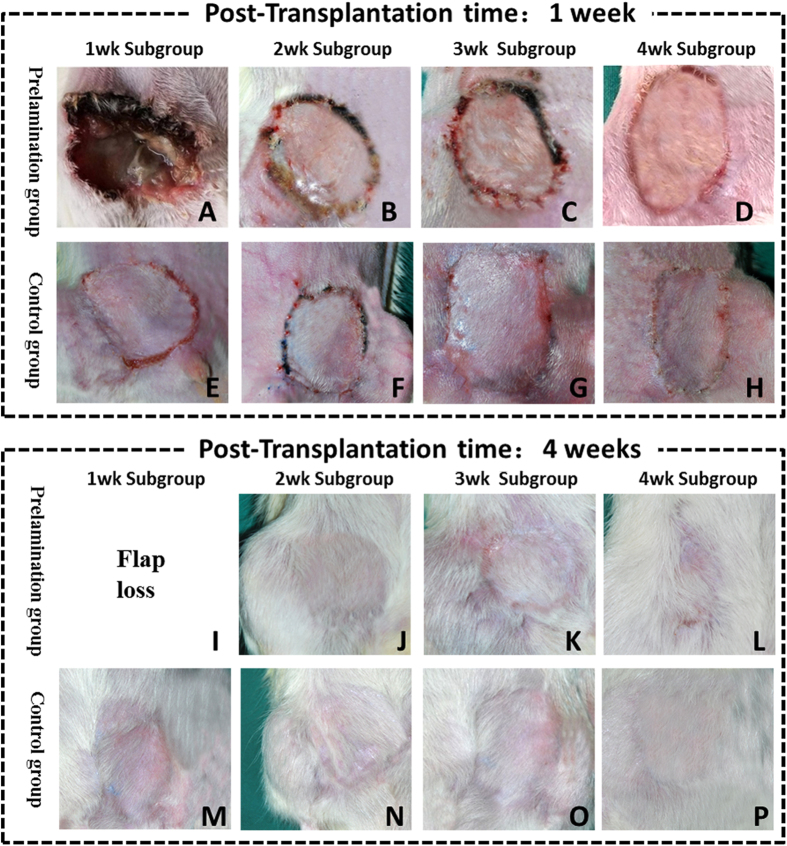
Gross view of transplanted skin flaps from each subgroup at post-transplantation weeks 1 and 4.

**Figure 5:**
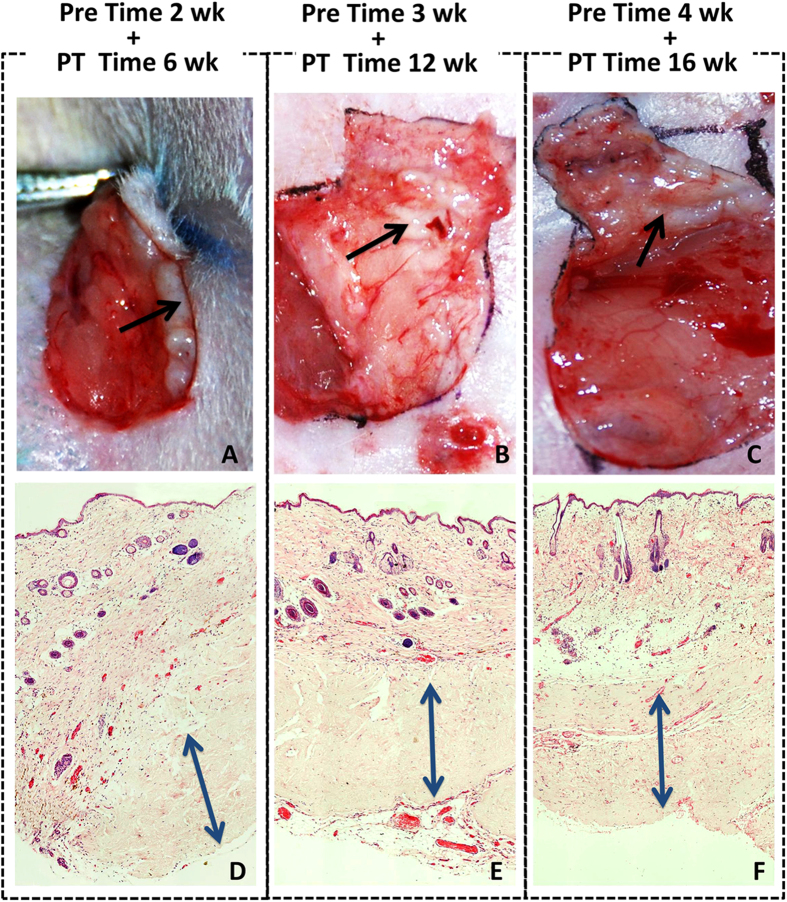
Long-term outcome of hADM. After *in vivo* incubation of 8 weeks (prelamination for 2 weeks, plus 6 weeks post-transplantation) (**A,D**), 15 weeks (3 plus 12 weeks) (**B,E**) and 20 weeks (4 plus 16 weeks) (**C,F**), hADM (arrow) appears as a pink-white patch well adhered to the adjacent host tissue. Histopathological evaluation demonstrates that hADM (double headed arrow) achieves sufficient cell infiltration and blood vessel formation and maintains its original architecture.

Animals were anesthetized with vaporized oxygen and isofluorane (Matrx VIP3000, Midmark, America). A 2.5 cm × 4.0 cm full-thickness skin wound was created in the right inguinal region, leaving the fascia intact. The removed skin and subcutaneous tissue was then trimmed into a thin split thickness skin graft for reconstruction (Figs 1A and 2C–F).

In the prelamination group, hADM was placed on the fascia and fixed in place with suture. An autologous split-thickness skin graft was overlaid on the hADM and fixed with a bolster dressing. In the control group, hADM was not added prior to the skin graft, but all other surgical manipulation was the same as in the prelamination group.

### Experimental Protocol

48 male Fisher 344 rats were randomly divided into even-sized groups as either controls or receiving prelaminated flaps (Fig. 1B).

Six rats in each prelamination subgroup were observed for 1, 2, 3 and 4 weeks, respectively. At these predetermined time points, histopathologic examination of the skin flaps was performed. Prelaminated skin flaps, based on the inferior epigastric artery, were raised and microsurgically transplanted back to the groin defect. Skin flap survival and hADM outcome were evaluated at post-transplantation weeks 8, 12, and 16.

All experiments in this study were performed in accordance with the Shanghai Jiaotong University School of Medicine Guide for Laboratory Animals. The protocol was approved by the Animal Care and Use Committee of Shanghai Ninth People’s Hospital. All efforts were made to minimize animal suffering.

### Gross and histopathological examination during skin flap prelamination

Bolster dressings were removed one week after prelamination. Skin flap size, survival, and integrity (presence of ulceration and/or necrosis) was assessed daily.

Full-thickness biopsies from prelaminated skin flaps were fixed in 4% paraformaldehyde overnight and then embedded in paraffin. 5-μm histological sections were prepared using standard protocols and stained with hematoxylin and eosin (H&E) and Masson’s trichrome. The histopathologic examination mainly focused on neovascularization, cellular infiltration of the hADM, and structural maintenance of the flaps.

For each H&E stain slide specimen, ten fields were randomly chosen to be viewed under high power at either the skin graft/hADM interface (prelamination group) or skin/fascia interface (control group). Vascularity was defined by the presence of structures exhibiting typical vascular walls and containing red blood cells; vessel count was calculated as the average number of blood vessels present in three sample sections^23^.

### Prelaminated skin flap and orthotopic microsurgical transplantation

#### Microangiography of related blood vessels and new flap

**Figure 6:**
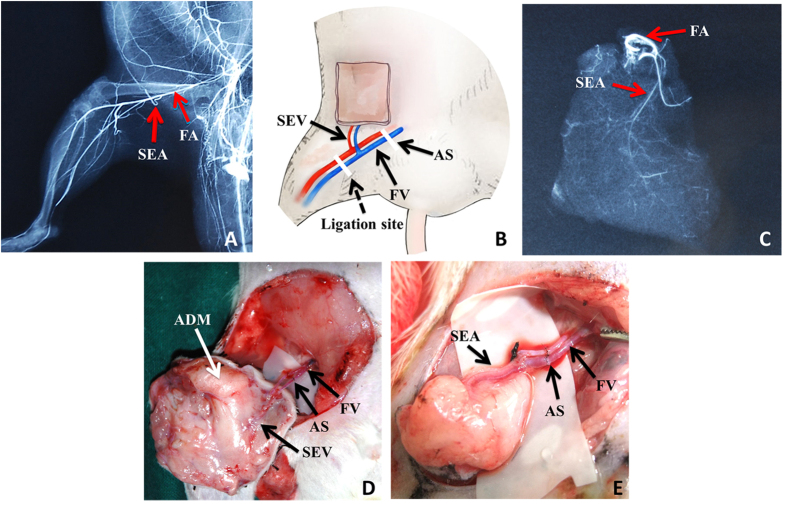
SEA-based prelaminated skin flap elevation and orthotopic microsurgical transplantation. (**A**) Microangiography of rat groin region and hind limb. The SEA arises from the femoral artery close to the site giving off the saphenous and popliteal arteries. (**B**) Schematic representation of SEA-based prelaminated flap. AS, anastomosis site. (**C**) Microangiography of a prelaminated flap demonstrates the flap was well supplied with SEA. (**D**) A SEA-based, prelaminated skin flap was raised and replanted to its original location with vascular anastomosis. hADM was well integrated with fascia and skin graft. (**E**) A SEA-based skin flap without hADM was also replanted.

Before transplantation, microangiography of the prelaminated skin flap and rat groin and hind limb was performed according to a previously described approach^24^ (Fig. 6).

#### Prelaminated skin flap elevation and microsurgical transplantation

Elevation and transplantation of prelaminated skin flaps was performed at either 1, 2, 3 or 4 weeks of prelamination.

To elevate the flaps, the original skin incision in the right groin was reopened, and previously implanted skin, hADM, and the basal inguinal fascia was elevated as a whole. The hADM appeared whitish pink. The superficial epigastric vessel (SEV) and femoral vessel (FV) were exposed and carefully dissected. The FV was clamped at both ends, and the distal end ligated and transected. After division of the proximal end of the FV, the SEV -based, prelaminated skin flap was harvested and flushed with cold heparinized lactated Ringer’s solution until the venous outflow became clear. Finally, end-to-end vascular anastomosis was performed at the proximal site of the FV to replant the skin flap back to the right inguinal region.

After surgery, rats were given 1 ml lactated Ringer’s solution subcutaneously to compensate for fluid loss during the surgery.

### Evaluation of free flap survival and long term outcome of ADM after orthotopic transplantation

The viability and integrity of transplanted skin flaps assessed daily. As described previously, full thickness biopsies from transplanted skin flaps at post-transplantation week 8, 12, and 16 were harvested for histopathological examination

### Statistical Analysis

All data were calculated as mean ± standard error, and significance was determined by performing Analysis of variance (ANOVA) (SPSS19.0 for Windows, SPSS, Inc., Chicago, IL). P values ≤ 0.05 were considered statistically significant.

## Additional Information

**How to cite this article**: Zhou, X. *et al*. A novel animal model for skin flap prelamination with biomaterials. *Sci. Rep*. **6**, 34144; doi: 10.1038/srep34144 (2016).

## Figures and Tables

**Table 1:** Skin flap engineering.

		Biomaterials category	
		Skin substitutes	Bioprosthetic mesh
Way to vascularization	Prefabrication	① Houle, human, lateral femoral circumflex artery + Integra, microsurgical repair^7^	① Erkin, rat, inferior epigastric + Alloderm, no repair^10^
		② Tanaka, rabbit, saphenous vessels + porcinetype I collagen sponge, microsurgical transplantation^8^	② Chung, rat, superficial epigastric vessels + Alloderm, pedicled repair^9^
			③ MacLeod, rat, superficial epigastric pedicles + Permacole, no repair^25^
	Prelamination	No report	This study, rat, superficial epigastric vessels based inguinal fascia + hADM, microsurgical repair
